# A re-sequencing based assessment of genomic heterogeneity and fast neutron-induced deletions in a common bean cultivar

**DOI:** 10.3389/fpls.2013.00210

**Published:** 2013-06-25

**Authors:** Jamie A. O'Rourke, Luis P. Iniguez, Bruna Bucciarelli, Jeffrey Roessler, Jeremy Schmutz, Phillip E. McClean, Scott A. Jackson, Georgina Hernandez, Michelle A. Graham, Robert M. Stupar, Carroll P. Vance

**Affiliations:** ^1^Plant Science Research Unit, USDA-Agricultural Research ServiceSt. Paul, MN, USA; ^2^Department of Agronomy and Plant Genetics, University of MinnesotaSt. Paul, MN, USA; ^3^Centro de Ciencias Genomicas-Universidad Nacional Autonoma de MexicoCuernavaca, Mexico; ^4^Hudson Alpha Institute for BiotechnologyHuntsville, AL, USA; ^5^Department of Plant Sciences, North Dakota State UniversityFargo, ND, USA; ^6^Department of Crop and Soil Sciences, University of GeorgiaAthens, GA, USA; ^7^Corn Insects and Crop Genetics Research Unit, USDA-Agricultural Research ServiceAmes, IA, USA

**Keywords:** *Phaseolus vulgaris*, common bean, natural variation, structural variation, fast neutron mutation, DNA-Seq

## Abstract

A small fast neutron (FN) mutant population has been established from *Phaseolus vulgaris* cv. Red Hawk. We leveraged the available *P. vulgaris* genome sequence and high throughput next generation DNA sequencing to examine the genomic structure of five *P. vulgaris* cv. Red Hawk FN mutants with striking visual phenotypes. Analysis of these genomes identified three classes of structural variation (SV); between cultivar variation, natural variation within the FN mutant population, and FN induced mutagenesis. Our analyses focused on the latter two classes. We identified 23 large deletions (>40 bp) common to multiple individuals, illustrating residual heterogeneity and regions of SV within the common bean cv. Red Hawk. An additional 18 large deletions were identified in individual mutant plants. These deletions, ranging in size from 40 bp to 43,000 bp, are potentially the result of FN mutagenesis. Six of the 18 deletions lie near or within gene coding regions, identifying potential candidate genes causing the mutant phenotype.

## Introduction

Common bean, *Phaseolus vulgaris* L., is an important source of proteins and carbohydrates for over three million people worldwide (Broughton et al., 2003). Despite its dietary importance, genetic resources for common bean have lagged behind those of “model legumes” soybean, *Medicago truncatula*, and *Lotus japonicus*. However, next generation sequencing (NGS) technologies now make genomic studies applicable to any species of interest.

Mutants are important tools in deciphering gene functions. In common bean, individual mutants can be created for a gene of interest through plant transformation (Aragao et al., 1996; Kwapata et al., 2012). Gene expression patterns of various genes in common bean can also be knocked out or down through the use of virus induced gene silencing (Diaz-Camino et al., 2011; Zhang et al., 2013). These methods both require prior knowledge of genes of interest. In contrast, mutant populations can be screened for phenotypes of interest and genes responsible identified through various approaches. The analysis of traits in various species including those related to plant architecture, yield, and stress response genes have been improved by utilizing mutant screens (Papdi et al., 2010; Bolon et al., 2011; Ma et al., 2013).

Structural variation (SV), including presence absence variation, inner and intra chromosomal translocations, insertions, and deletions is believed to be an important component of phenotypic diversity in both plants and animals (Lai et al., 2010; Stankiewicz and Lupski, 2010; Cao et al., 2011; Eichten et al., 2011; Wang et al., 2011; McHale et al., 2012). Genomic variation both between and within cultivars has been identified in soybean (Bolon et al., 2011; Haun et al., 2011; McHale et al., 2012), maize (Lai et al., 2010), rice (Huang et al., 2012), and Arabidopsis (Cao et al., 2011; Belfield et al., 2012). Two studies in soybean examining SV between cultivars (McHale et al., 2012) and within the Williams 82 cultivar (Haun et al., 2011) determined the genomic regions most enriched for SV were gene rich regions, particularly regions containing resistance genes.

Here, we present a small fast neutron (FN) mutant population for common bean and demonstrate how NGS technologies, such as DNA-seq provide for fast, high quality analysis of genomic variation to identify potential candidate genes for observed phenotypes. Additionally, the DNA-seq data allowed us to examine the natural variation existing within the Red Hawk cultivar. Such natural variation is a rich source of genomic diversity that can be utilized in future cultivar development.

## Materials and methods

### Development of *Phaseolus vulgaris* fast neutron population

Ten thousand *P. vulgaris* cv. Red Hawk seeds (Kelly et al., 1998), an Andean cultivar adapted for growth in the upper Midwest, were sent to the McClellan Nuclear Radiation Center at the University of California-Davis for irradiation. Five thousand seeds were treated with either 16 or 32 Gys of FN radiation. Treated seeds were sent to the Illinois Crop Improvement Association (ICIA) facility in Puerto Rico in November 2009 along with 200 wild type seeds from the same seed lot. Approximately 70% of the 5000 seeds treated with 16 Gys germinated, while none of the seeds treated with 32 Gys germinated. Seedlings were allowed to mature at the ICIA facility, where plants were phenotyped and seeds were collected from all mature plants. Seeds from ~88 plants with striking mutant phenotypes such as developmental delays, plant stature, pod set, and pod size variations, were harvested individually. Remaining mutant plants were bulk harvested. Wild type plants grown at ICIA were also bulk harvested.

Seeds from individually collected plants were planted at the University of Minnesota Experiment Station in St. Paul in 2010. Approximately 10,000 seeds from the bulk collection of mutants were also planted. Phenotyping was performed throughout the growth season, complemented by photographs. Selected individuals with visible and/or maturity phenotype variations were harvested. In 2011, seeds from selected 2010 M2 individuals were planted in 10 ft rows (~20 seeds). Phenotypes observed throughout the 2011 growth season were compared to documented phenotypes from previous years to determine if trait expression was consistent. Additionally, segregation among the 20 plants per mutant line was noted. Three to four individuals in each row with visible/stable traits were tagged, photographed and seed was harvested.

### DNA-seq analysis of fast neutron mutants

**Figure 1:**
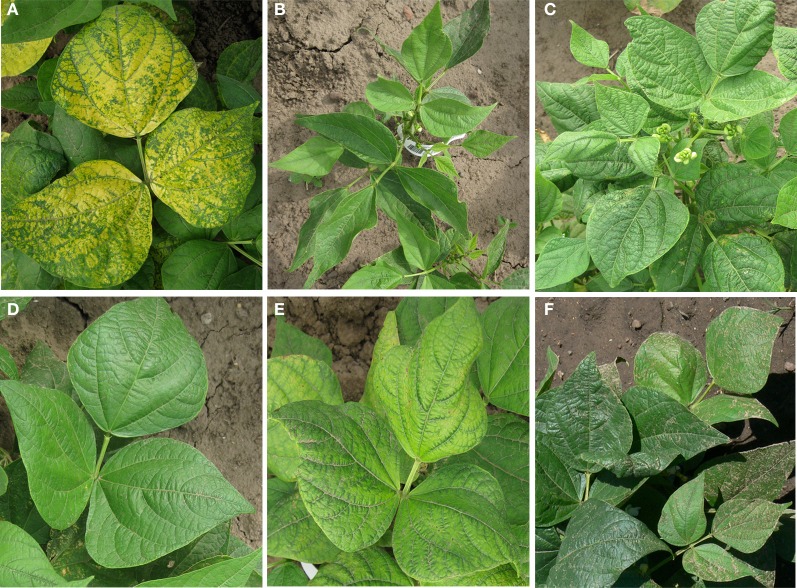
**Visual phenotype of five *Phaseolus vulgaris* cv. Red Hawk mutants from the fast neutron mutant population chosen for DNA-seq**. All plants are from the M2 generation and were grown at the University of Minnesota Experiment Station in St. Paul, MN in 2011. **(A)** Mutant 1R5C01r5CPVMN11 referred to as decorative due to the chlorotic patterning on leaves early in the growing season. **(B)** Mutant 1R19C15r28CPVMN11 referred to as lanceolate due to the elongated shape of the leaf. This mutant also appeared shorter than the WT plants in the field. **(C)** Mutant 1R22C04r31CPVMN11 referred to as rugose due to the crinkled leaf texture. The petioles of this plant also appeared shorter and more upright than the WT. **(D)** Mutant 2R29C12r78CPVMN11 referred to as the maturity mutant. This plant is phenotypically identical to the WT except for a delay in maturity. **(E)** Mutant 3R5C25r87CPVMN11 referred to as the chlorotic mutant due to the interveinal chlorosis patterning observed in the leaves. **(F)** Wild-type *Phaseolus vulgaris* cv. Red Hawk for comparison.

Five FN mutant plants with different, stable, obvious phenotypes (Figure 1) were chosen for paired end sequence analysis. The following FN mutant plants chosen: 1R5C01r5CPVMN11, a plant with decorative chlorotic leaves early in the growing season (Figure 1A), 1R19C15r28CPVMN11, a small plant with lanceolate leaves (Figure 1B); 1R22C04r31CPVMN11, an upright plant with rugose leaves (Figure 1C); 2R29C12r78CPVMN11; which phenotypically resembled the wild type plant but was delayed in maturity (Figure 1D); and 3R5C25r87CPVMN11, which exhibited interveinal chlorosis (Figure 1E). The mutant plants will respectively be referred to as lanceolate, rugose, decorative, maturity, and chlorotic throughout the rest of the manuscript. M3 seeds of the plants chosen for sequencing were collected and planted at the University of Minnesota Experiment Station in St. Paul in 2012 to ensure the phenotype was maintained through the M3 generation. Leaf tissue from a representative wild type plant and from each of the chosen mutant plants at the M2 generation was collected from 2011 field-grown plants early in the morning and immediately placed at −80°C to inhibit DNA degradation. DNA from all six plants (WT and five mutants) was extracted using the phenol:chloroform method as described (Liu et al., 1997). Each DNA sample was visually inspected on a 1% agarose gel, to ensure that the samples were not degraded. DNA concentration and purity was assessed using an Agilent 2100® Bioanalyzer™ (Agilent®, Santa Clara, CA). DNA samples were submitted to the molecular biology core at the Mayo Clinic, Rochester, MN for paired end sequencing on an Illumina HiSeq 2000. To reduce variability, DNA from all samples were multiplexed and run in a single lane. Low quality reads and adaptor sequences were removed, resulting in 31 million paired end reads per sample.

**Figure 2:**
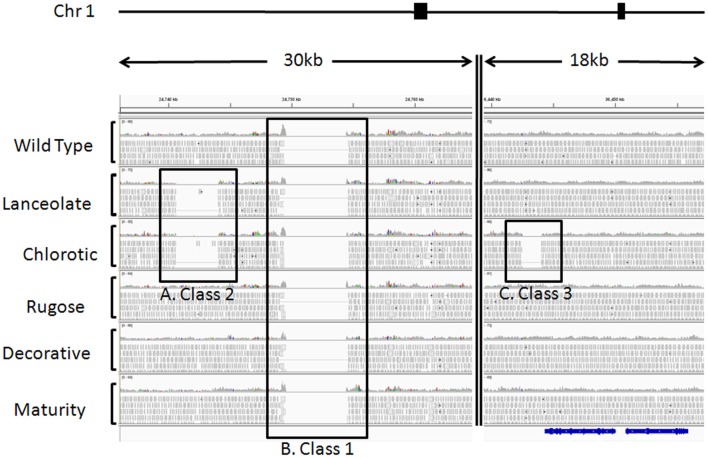
**Example of sequence alignments illustrating cultivar heterogeneity and fast neutron induced deletions**. Paired end sequences from six Red Hawk individuals were aligned to the G 19833 genome sequence available at www.phytozome.net using BWA (Li and Durbin, 2009) with default parameters. Using CREST and custom perl scripts, we identified statistically significant deletions illustrating three classes of SV. SV was visualized using IGV (Robinson et al., 2011). These regions of SV were all identified on chromosome 1, from the two regions highlighted by filled black boxes above the alignments. The double vertical line within the alignments represents chromosomal region between the two genomic regions depicted. For each individual, a histogram plot illustrates the read depth with individual reads plotted below. Black boxes highlight the three classes of SV. **(A)** Class 2, regions of genomic heterogeneity within the Red Hawk cultivar. These deletions were identified in two or more Red Hawk individuals and represent the residual heterogeneity in the fast neutron mutant population. This deletion spans 3408 bp on chromosome 1 and is only evident in the lanceolate and chlorotic individuals. **(B)** Class 1, sequences present in the reference genome, but missing in all Red Hawk individuals. These regions illustrate the differences between the common bean cultivars. This particular region spans 5000 bp of the reference genome. **(C)** Class 3, sequences missing in a single individual but present in all other lines. This class is most likely the result of fast neutron mutagenesis and may be responsible for the mutant phenotype. This particular deletion is approximately 1500 bp long in the chlorotic mutant and is immediately downstream of the predicted gene Phvul.001G128600 (shown in blue below alignments), which encodes a RecA protein.

**Figure 3:**
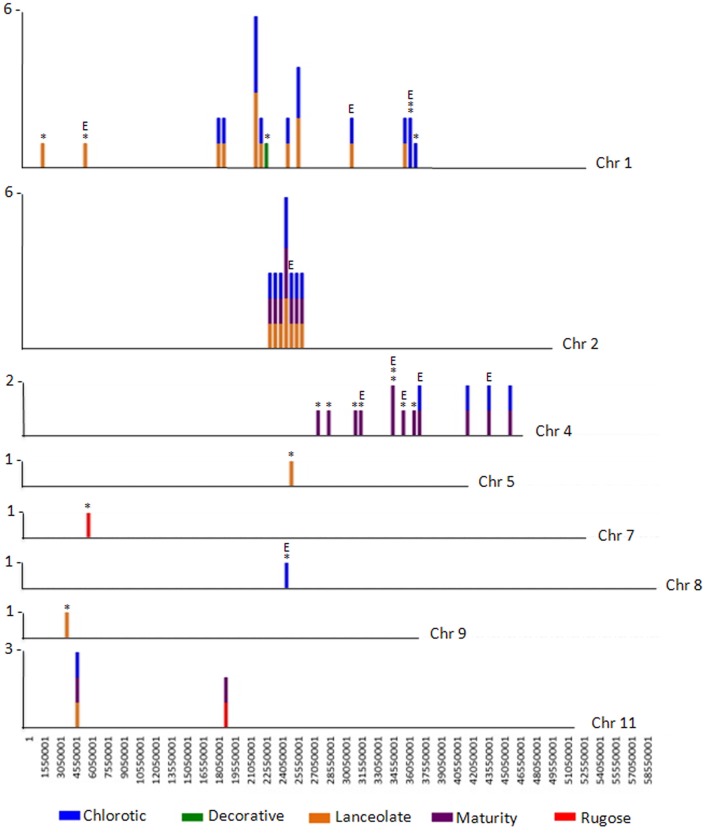
**CREST analyses identifies SV belonging to Class 2 and Class 3**. The number of large genomic deletions in each class identified per 500,000 bp on each chromosome of *P. vulgaris* were counted and plotted as a vertical line. The height of the line indicates the number of SV per 500,000 bp region that were identified. The largest number of SV per chromosomal region is noted to the left of each chromosome. The mutant containing the deletion is represented by color: blue, chlorotic; purple, maturity; green, decorative; red, rugose; orange, lanceolate. Deletions belonging to Class 3 are likely a result of FN mutagenesis and are highlighted by an asterisk (*). Deletions potentially impacting gene expression are highlighted with an E. Note the region of natural variation (SV Class 2) shared by three mutant plants on chromosome 2.

Paired-end genome sequences were mapped to the *P. vulgaris* G 19833 genome sequence available at www.phytozome.net using BWA (Li and Durbin, 2009) with default parameters. The resulting mapping files were further sorted, indexed, and translated to binary format (BAM files) using samtools (Li et al., 2009). The sequence alignments were visualized using IGV (Robinson et al., 2011). This approach aligned 70% of all Red Hawk DNA sequences to 88% of each of the 11 *P. vulgaris* chromosomes with 12X sequence depth. Regions of genomic deletions were identified using custom perl scripts. To confirm these deletions, the program, CREST (Wang et al., 2011) was also used to screen the FN mutant plants. This program identifies genomic deletions, insertions, inversions, and translocations by identifying soft clipped reads and the read coverage at potential breakpoints to calculate if the probability of observing the number of soft clipped reads at a given location is >0.05 based on a binomial distribution. Statistically significant (*P* < 0.05) SV are retained for further consideration. CREST analysis was performed for each mutant compared to the wild type control. Genomic deletions resulting from differences between cultivars were masked using the –*g* function. We chose to focus our analysis on genomic deletions as these are called with the greatest confidence and can be confirmed by visual screening of genomic alignments. Deletions >40 bp identified by both perl scripts and CREST analysis were further characterized by genic location: intergenic, promoter, exon, intron, and 3′UTR. Genomic regions of natural variance within the cultivar were identified by identifying deletions common to multiple, but not all, FN mutant plants (Figures 2A, 3). Single nucleotide polymorphisms (SNPs), small (<40 bp) insertions and deletions (INDELs) (either unique to a single plant or common to multiple plants) were identified using the pileup function in SAMtools (Li et al., 2009). Unique or common SNPs and INDELs were identified using the compareBed function of BEDtools (Quinlan and Hall, 2010) and custom perl scripts. SNPs and INDELs with a read depth <6 (half the average genome coverage) were removed from further analysis. Custom perl scripts were used to characterize SNPs and INDELs by genic locations as described above.

## Results

### Mutant collection

FN mutant populations have proven to be a valuable asset for genetic studies in a variety of crop species (Starker et al., 2006; Bolon et al., 2011; Xiao et al., 2011). We have established a small FN mutant population for common bean using *P. vulgaris* cv. Red Hawk. Seeds from 88 plants with stable, visual phenotypes are available for public use (Table 1) by contacting Dr. Carroll Vance at vance004@umn.edu or Jeff Roesler at roess001@umn.edu. Phenotypes observed in the field include varying degrees and types of chlorosis and altered maturity (usually delayed). Additionally, bulk seed from M2 and M3 plants is available for researchers wishing to screen mutants for a particular trait of interest.

**Table 1:** ***Phaseolus vulgaris* cv. Red Hawk fast neutron mutant population**.

**Plant ID**	**2010 Phenotype**	**2011 Phenotype**	**Mat**	**M3 Seg**	**Generation**
1R04C18r4CPVMN11	Erect growth	Erect growth	N	N	M3
1R05C01r5CPVMN11	Stunted dwarf, slt chlorotic	Stunted dwarf, slt chlorotic	L	N	M3
1R06C05r6CPVMN11	Stunted, delayed	Stunted	N	N	M3
1R06C09r7CPVMN11	Large leaves, chlorotic	WT pheno	E	N	M3
1R07C04r8CPVMN11	Stunted, delayed	Stunted, delayed	L	N	M3
1R07C11r9CPVMN11	Stunted, delayed	Stunted, delayed	L	N	M3
1R07C14r11CPVMN11	Stunted, delayed	Delayed growth	L	N	M3
1R09C01r13CPVMN11	Few leaves, flowering	WT pheno	N	N	M3
1R10C08r14CPVMN11	Delayed growth	Chlorotic	N	N	M3
1R11C15r16CPVMN11	Lanceolate leaves	Lanceolate leaves	L	N	M3
1R13C06r18CPVMN11	Few leaves, flowering	WT pheno	N	N	M3
1R14C12r20CPVMN11	Late maturity	Small plant	L	1:1	M3
1R14C33r21CPVMN11	Light green leaves	Light green leaves	N	N	M3
1R15C05r22CPVMN11	Stunted	Stunted	L	3:1	M3
1R15C10r23CPVMN11	Few leaves	WT pheno, note maturity	E	N	M3
1R16C03r24CPVMN10	Erect growth	WT pheno	N	N	M3
1R16C09r25CPVMN11	Few leaves	WT pheno	N	N	M3
1R17C13r26CPVMN11	Erect, large leaves	Erect, large leaves	N	N	M3
1R18C18r27CPVMN11	Tall, bushy, erect	WT pheno	N	N	M3
1R19C15r28CPVMN11	Lanceolate leaves	Lanceolate leaves, long petioles, some chlorosis	L	N	M3
1R20C06r29CPVMN11	Few leaves, flowering	WT pheno	N	N	M3
1R20C08r30CPVMN11	Delayed growth	WT pheno, note maturity	E	N	M3
1R22C04r31CPVMN11	Delayed growth	Rugose, stunted	L	N	M3
1R22C15r32CPVMN11	Leaf size and shape	Large leaves, long petioles	N	N	M3
1R22C27r33CPVMN11	Large leaves	Bushy, lots of leaves	N	N	M3
1R22C37r34CPVMN11	Leaf size and shape	WT pheno	N	N	M3
1R23C21r35CPVMN11	Small leaves	Lanceolate leaves, long petioles, some chlorosis	N	N	M3
1R24C13r37CPVMN11	Large leaves	Large leaves	E	N	M3
1R25C20r39CPVMN11	Lacks apical dominance, sprawling	Lacks apical dominance, sprawling	N	1:5	M3
1R26C07r40CPVMN11	Bushy	Bushy	N	N	M3
1R26C24r41CPVMN11	Lanceolate leaves	WT pheno, note maturity	E	N	M3
1R26C26r42CPVMN11	Stunted	Stunted	L	N	M3
1R29C03r46CPVMN11	Bushy	Bushy	N	1:1	M3
1R30C02r47CPVMN11	Chlorotic	Chlorotic	L	N	M3
1R30C06r48CPVMN11	Large leaves	Large leaves	N	N	M3
1R31C13r49CPVMN11	Few leaves, flowering	WT pheno, note maturity	E	N	M3
1R31C28r50CPVMN11	Few leaves, flowering	Few Leaves	N	N	M3
1R32C06r51CPVMN11	Late maturity	Late maturity	L	N	M3
1R32C11r52CPVMN11	Short plant	Short plant	N	N	M3
1R32C17r53CPVMN11	Bushy	Bushy	N	N	M3
1R33C17r54CPVMN11	Large, lanceolate leaves	Large, lanceolate leaves	N	N	M3
1R33C32r55CPVMN11	Short, bushy and slightly rugose	Short, bushy	N	N	M3
1R35C27r56CPVMN11	Tall and bushy	Bushy	N	1:1	M3
1R37C06r57CPVMN11	Bushy and leaf size varies	Bushy	L	N	M3
1R37C19r58CPVMN11	Many leaves fused to form unifoliate	Long petioles	N	N	M3
1R41C06r60CPVMN11	Rugose	Fewer pods	L	N	M3
1R41C22r61CPVMN11	Erect growth, light green chlorotic leaves	Erect growth, light green chlorotic leaves	N	N	M3
1R42C02r62CPVMN11	Small chlorotic plant	Small chlorotic plant	N	1:2	M3
1R43C24r63CPVMN11	Wavy curled leaves	WT pheno, note maturity	L	N	M3
1R44C20r64CPVMN11	Bushy, wavy and curled leaves	Bushy, wavy and curled leaves	E	N	M3
2R14C01r66CPVMN11	Stunted, cupped leaves and few flowers	Stunted, cupped leaves and few flowers	L	N	M3
2R14C13r67CPVMN11	Stunted, rugose curled leaves, short petioles	WT pheno	N	N	M3
2R18C06r69CPVMN11	Short, stunted, delayed	Erect	L	2:1	M3
2R24C27r73CPVMN10	Short compact plant	Short compact plant	N	N	M3
2R25C11r74CPVMN11	Slight chlorotic	Erect growth	N	N	M3
2R26C31r75CPVMN11	Slight chlorotic	Slight chlorotic	N	N	M3
2R27C18r76CPVMN11	Viney, spindly, few leaves, large cupped leaves	Viney, spindly, few leaves, large cupped leaves	L	N	M3
2R27C20r77CPVMN11	Lanceolate leaves	Bushy	N	N	M3
2R29C12r78CPVMN11	Few large leaves	WT pheno, note maturity	L	1:2	M3
2R33C02r80CPVMN11	Lanceolate curled leaves, very few flowers, late maturity	Curled leaves	L	N	M3
2R43C07r83CPVMN11	Large leaves, some fused trifoliates	Bushy	N	N	M3
2R43C38r84CPVMN11	Large dark green rugose leaves	WT pheno, note maturity	L	N	M3
3R04C09r85CPVMN11	Large leaves	Long petioles, a bit bushy	N	N	M3
3R05C13r86CPVMN11	Late maturity	Late maturity	L	N	M3
3R05C25r87CPVMN11	Slight chlorotic	Interveinal chlorosis	L	N	M3
3R06C13r89CPVMN11	Large cupped leaves	Slightly curled leaves	L	N	M3
3R07C22r90CPVMN11	Tall, erect growth	fewer pods, note maturity	L	N	M3
3R11C14r91CPVMN11	Large rugose leaves	rugose, spindly, very few pods	N	N	M3
3R16C03r92CPVMN11	Curled lanceolate leaves	Curled lanceolate leaves	L	N	M3
3R17C22r93CPVMN11	rugose, short petioles	rugose, short petioles	L	N	M3
R06C05CPVMN11	N/A	Short, curled leaves, spotty chlorosis	N/A	N/A	M2
R09C05CPVMN11	N/A	Few lateral branches, erect growth	N/A	N/A	M2
R10C09CPVMN11	N/A	Spindly, small leaves	N/A	N/A	M2
R11C21CPVMN11	N/A	Large leaves, odd nodes, rugose, many small branches	N/A	N/A	M2
R13C09CPVMN11	N/A	Short, rugose curled leaves	N/A	N/A	M2
R15C12CPVMN11	N/A	Short, pointed leaves	N/A	N/A	M2
R16C12CPVMN11	N/A	Short, bushy, lacks apical dominance, many small branches, small leaves	N/A	N/A	M2
R19C22CPVMN11	N/A	Spotty chlorosis like row 5 in M3 line	N/A	N/A	M2
R21C05CPVMN11	N/A	Short petioles, large rugose curled leaves	N/A	N/A	M2
R22C06CPVMN11	N/A	Tall, very long petioles	N/A	N/A	M2
R24C05CPVMN11	N/A	Few lateral branches and flowers	N/A	N/A	M2
R24C18CPVMN11	N/A	Stunted, small curled leaves	N/A	N/A	M2
R25C15CPVMN11	N/A	Very stunted mini-plant	N/A	N/A	M2
R28C12CPVMN11	N/A	Spindly, erect growth, small leaves	N/A	N/A	M2
R30C08CPVMN11	N/A	Stunted, few branches, no flowers	N/A	N/A	M2
R40C08CPVMN11	N/A	Stunted, small pointed leaves	N/A	N/A	M2
R47C24CPVMN11	N/A	Short, short petioles, small leaves	N/A	N/A	M2
R47C25CPVMN11	N/A	Short, chlorotic, pointed leaves	N/A	N/A	M2

Mat, Maturity; E, earlier than WT; N, normal; L, later than WT; M3 Seg, if M3 row planting is segregating ratio is noted. Generation, mutant generation seeds available.

### Use of DNA-seq to identify regions of structural variation

The DNA-seq reads from the five FN mutant lines and one wild-type cv. Red Hawk individual were mapped to the common bean reference genome sequence (accession G 19833). This analysis allowed us to identify three classes of SV (Figure 2): (1) sequences present in the reference genome sequence, but absent from all six of the Red Hawk individuals (Figure 2B); (2) sequences present in both the reference genome sequence and at least one Red Hawk individual, but absent in two or more Red Hawk individuals (Figure 2A); (3) sequences absent from one FN line, but present in all other samples (Figure 2C).

The Class 1 group primarily represents intra-specific structural genomic differences between accession G 19833 and cv. Red Hawk. We identified over one-thousand of these regions, in which all six Red Hawk individuals were missing >100 bp that is present in the reference genome. This is an interesting group of SV to catalog and may have important implications for understanding inter-cultivar phenotypic variation. However, these features do not inform our understanding of the mutant phenotypes in the FN lines. Therefore, we chose to focus the data analysis on the Class 2 and Class 3 groups, which exhibited structural polymorphism among the FN individuals.

The Class 2 group consists of DNA segments present in some FN individuals, but missing in at least two others (Figure 2A). It is highly unlikely the same genomic regions would be deleted in multiple plants by FN irradiation. Our analysis identified 24 genomic deletions belonging to Class 2, which illustrates the natural variation within the common bean cultivar Red Hawk. Specifically, ten unique deletions (*P* < 0.05) are shared by the lanceolate and chlorotic mutants on chromosome 1, nine genomic deletions were identified on chromosomes 2 and 11 in three mutant plants (lanceolate, maturity, and chlorotic), four deletions on chromosome 4 are common to the maturity and chlorotic mutants, and one deletion on chromosome 11 is shared by the rugose and maturity mutants (Figure 3, Table 2). Class 2 deletions range in size from 41 base pairs (bp) to 12,111 bp. Of all of these deletions, two are within gene introns and two are within 1000 bp upstream of the start codon or 1000 bp downstream of the stop codon, regions involved in regulating gene expression patterns (Table 2). All Class 2 deletions on chromosome 2 lie within 2.7 million bps of each other. This is a region exhibiting high heterogeneity (Figure 3). Within this region three individual FN lines share eight Class 2 deletions. The region is unaffected in the remaining two FN lines and the wild type plant.

**Table 2:** **Regions of natural structural variation identified within the cultivar, Red Hawk**.

**Chr**	**Deletion start (bp)**	**Deletion stop (bp)**	**Deletion size (bp)**	**Mutants with deletion**	**Genes potentially affected**	**Deletion relative to gene**
Chr01	18253164	18253570	406	Lanceolate, Chlorotic		
Chr01	19119759	19130873	11,114	Lanceolate, Chlorotic		
Chr01	21478079	2147855	376	Lanceolate, Chlorotic		
Chr01	21604603	21604792	189	Lanceolate, Chlorotic		
Chr01	22370255	22370296	41	Lanceolate, Chlorotic		
Chr01	24740621	24744029	3,408	Lanceolate, Chlorotic		
Chr01	25792230	25792287	57	Lanceolate, Chlorotic		
Chr01	25873578	25880798	7,220	Lanceolate, Chlorotic		
Chr01	30779938	30780112	174	Lanceolate, Chlorotic	Phvul.001G111700	P
Chr01	35937306	35938111	805	Lanceolate, Chlorotic		
Chr02	23462357	23462602	245	Lanceolate, Maturity, Chlorotic		
Chr02	23744872	23744518	646	Lanceolate, Maturity, Chlorotic		
Chr02	24500720	24504943	4,223	Lanceolate, Maturity, Chlorotic		
Chr02	24718765	24718893	128	Lanceolate, Maturity, Chlorotic		
Chr02	24728318	24728663	345	Lanceolate, Maturity, Chlorotic		
Chr02	25130420	25130510	90	Lanceolate, Maturity, Chlorotic	Phvul.002G125100	I
Chr02	25611051	25613798	2,747	Lanceolate, Maturity, Chlorotic		
Chr02	26229269	26229410	141	Lanceolate, Maturity, Chlorotic		
Chr04	3745720	3746721	1,001	Matruity, Chlorotic	Phvul.004G33700	I
Chr04	4160322	4160726	404	Matruity, Chlorotic		
Chr04	4374139	4386350	12,111	Matruity, Chlorotic	Phvul.004G039400	P
					Phvul.004G039500	D
Chr04	4571494	4575913	4,419	Matruity, Chlorotic		
Chr11	5406621	5407835	1,214	Lanceolate, Maturity, Chlorotic		
Chr11	19639745	19639812	67	Rugose, Maturity		

Genomic deletions identified by the program CREST that belong to SV Class 2. All SV were visually verified using IGV. This class of SV represents regions of heterogeneity within Red Hawk.

P, Deletion is within 1000 bp of start codon; I, Deletion is in an intron; D, Deletion is within 1000 bp of stop codon.

The remaining 18 deletions identified by CREST (*P* < 0.05) belong to Class 3. This class is composed of sequences absent from a single FN line, but present in all other samples (Figure 2C, Table 3). Deletions belonging to Class 3 range in size from 41 to over 43,000 bp and are found on chromosomes 1, 4, 5, 7, 8, and 9. Twelve of the eighteen Class 3 deletions are in intergenic regions of the genome, though as genome annotation improves these regions may contain as of yet unpredicted genes. Six deletions belonging to Class 3 have the potential to alter gene expression patterns. Regions immediately upstream or downstream of gene coding regions are likely involved in regulating gene expression. Three deletions are located within 1000 bp of gene start or stop codons of Phvul.001G128600, Phvul.004G029200, Phvul.004G031900, and Phvul.004G032000 in the chlorotic and maturity mutants. The latter two genes are tightly linked in the genome, so a single deletion may affect the expression of multiple genes. Two Class 3 deletions shorten the introns of Phvul.001G050100 in the lanceolate mutant and Phvul.004G031200 in the maturity mutant. Finally, a 43,034 bp deletion on chromosome 8 in the chlorotic mutant removes the entire sequence for Phvul.008G141500. In summary, we were able to identify statistically significant SV belonging to Class 3 within either the coding region or the potential regulatory region of predicted genes for three of the five mutant plants (lanceolate, maturity, and chlorotic). Genes likely impacted by these deletions represent the most likely candidates responsible for the mutant phenotype of the lanceolate, maturity, and chlorotic mutants. For decorative and rugose, our analysis pipeline failed to identify Class 3 deletions within the regulatory or coding region of genes. These phenotypes may be a result of a heterozygous deletion, a small (<40 bp) INDEL, or a SNP.

**Table 3:** **Putative fast neutron induced genomic deletions**.

**Chromosome**	**Deletion start (bp)**	**Deletion stop (bp)**	**Deletion size (bp)**	**Mutant with deletion**	**Genes potentially affected**	**Deletion relative to gene**
Chr01	1478816	1481247	2,431	Lanceolate		
Chr01	5557900	5558034	134	Lanceolate	Phvul.001G050100	I
Chr01	22818248	22818444	196	Decorative		
Chr01	36310535	36310624	89	Chlorotic		
Chr01	36442517	36440305	1,518	Chlorotic	Phvul.001G128600	D
Chr01	36556650	36556691	41	Chlorotic		
Chr04	2782039	2782294	255	Maturity		
Chr04	2897805	2898208	403	Maturity		
Chr04	3115668	3116070	402	Maturity		
Chr04	3167395	3168878	1,483	Maturity	Phvul.004G029200	P
Chr04	3474839	3481382	6,543	Maturity		
Chr04	3489741	3490354	613	Maturity	Phvul.004G031300	I
Chr04	3568924	3569058	134	Maturity	Phvul.004G031900	D
					Phvul.004G032000	D
Chr04	3679845	3684349	4,504	Maturity		
Chr05	25018705	25018771	66	Lanceolate		
Chr07	6354277	6354449	2,762	Lanceolate		
Chr08	24523869	24566903	43,034	Chlorotic	Phvul.008G141500	G
Chr09	431942	434704	2,762	Lanceolate		

Class 3 SV identified by the program CREST and visually verified using IGV. This SV class identifies sequences absent from one FN line, but present in all other samples. This class of SV is most likely a result of FN mutagenesis.

P, Deletion is within 1000 bp of start codon; I, Deletion is in an intron; D, Deletion is within 1000 bp of stop codon; G, whole gene deleted.

DNA-seq permits genome analyses on a base pair scale. Using the approach described in the materials and methods we estimated that there are 32,499 SNPs and 20,363 INDELs shared by multiple FN lines, most likely representing the natural variation caused by genetic heterogeneity within the Red Hawk cultivar (Class 2). We also estimated 92,205 SNPS and 20,340 INDELs unique to a single FN line (Class 3). As described earlier, Class 3 SNPs and INDELs are most likely a result of the FN mutagenesis. Both Class 2 and Class 3 SNPs and INDELs were mapped to the available *P. vulgaris* genome to determine whether the change in genomic architecture corresponded to a genic region (Tables 4, 5). As was observed with the larger deletions, the majority of the INDELs and SNPs mapped to intergenic regions. Confirmation by PCR will be necessary to determine the false discovery rate of the SNP and INDEL identification.

**Table 4:** **Identification and classification of SNPs in the five mutant plants**.

**Number of mutants with SNP**	**SV class**	**SNPs in intergenic regions**	**SNPs within gene (promoter, exon, intron)**
SNPs unique to 1 plant	3	74,534	17,761
SNPs shared by 2 plants	2	14,515	2,376
SNPs shared by 3 plants	2	7,190	1,073
SNPs shared by 4 plants	2	4,159	744
SNPs shared by 5 plants	2	2,033	409

SNPs shared by more than one mutant plant (Class 2) represent natural variation existing in the Red Hawk cultivar. SNPs unique to a single mutant (Class 3) are likely caused by FN mutagenesis. SNPs within a gene region are more likely to impact gene expression patterns than those in intergenic regions.

**Table 5:** **Identification and classification of INDELs in the five mutant plants**.

**Number of mutants with INDEL**	**SV class**	**INDELs in intergenic regions**	**Indels within a gene (promoter, exon, intron)**
INDELs unique to 1 plant	3	17,606	2,704
INDELs shared by 2 plants	2	8,042	1,204
INDELs shared by 3 plants	2	4,771	740
INDELs shared by 4 plants	2	2,877	445
INDELs shared by 5 plants	2	1,977	271

INDELs shared by more than one mutant plant likely represent natural variation existing in the Red Hawk cultivar (Class 2) while INDELs unqiue to a single mutant (Class 3) are likely a result of FN mutagenesis. INDELs within a gene region are more likely to impact gene expression patterns than those in intergenic regions.

## Discussion

We demonstrate the feasibility of utilizing high throughput DNA sequencing to analyze FN mutant plants. The use of high throughput sequencing allows the scale of our analysis to be reduced to a base-pair level, providing for the identification and analysis of SNPs, indels, and larger genomic deletions using a single experimental platform. Our analysis identified SV in each Red Hawk individual (wild type and FN mutants) in comparison to the *P. vulgaris* (accession G 19833) reference genome sequence (available at www.phytozome.net). These SV are inferred to be sequences lost (deleted) from the respective Red Hawk individuals or sequences recently gained by G 19833. (Identifying sequences missing in G 19833 that are present in Red Hawk requires a *de-novo* assembly of the Red Hawk genome, which is beyond the scope of this analysis.) One may assume that the majority of the SV is either natural or FN-induced deletions in Red Hawk; therefore these events will be referred to as “deletions” throughout the discussion.

Our analysis identified three classes of SV (Figure 2). Class 1 represents the putative inter-cultivar SV, large sequence segments that are missing from all sequenced Red Hawk individuals in this study, but are present in the current G 19833 assembly (Figure 2B). Class 2 represents the intra-cultivar SV exhibiting differences among Red Hawk individuals (Figure 2A). Class 3 represents SV specific to a single Red Hawk individual, which were potentially generated by the FN irradiation (Figure 2C). We focused our analysis on the 23 Class 2 and 18 Class 3 SV that were identified in this analysis. However, it is important to recognize that these classifications are tentative, as a deeper sampling of Red Hawk individuals may re-classify some variants. For example, the limited sampling of one wild-type and five mutated Red Hawk individuals suggests that some of our Class 3 SV may be low frequency natural variants that would have been identified in more than one Red Hawk individual (and thereby be a Class 2 SV) if a larger number of genotypes had been sampled. Similarly, some Class 1 SV may be present at low frequency in Red Hawk, suggesting that these would be re-classified as Class 2 SV in a deeper sampling of genotypes. It is probable that increasing the number of sequenced individuals from Red Hawk and/or G 19833 individuals would identify many additional Class 1 and Class 2 SV, and strengthen the confidence of the Class 3 calls.

A particularly interesting heterogeneous mixture of deletions was identified in a 2.7 million bp region on chromosome 2 of Red Hawk (Figure 3), and represents an intriguing cluster of Class 2 SV. Sequence analysis of the 159 genes within this region revealed 21 are involved in disease resistance response (one dirigent like protein, six leucine rich repeat proteins, and 14 NB-ARC-LRR domain containing disease resistance proteins). This is consistent with previous studies which identified an over-abundance of disease resistance genes located within regions of natural variation (McHale et al., 2012). Additional regions of Class 2 SV are apparent on chromosomes 1, 4, and 11. Analysis of the DNA-seq data for the five individual FN lines suggests the level of natural variation present within the Red Hawk cultivar is higher than that induced by FN mutagenesis. However, aside from the region on chromosome 2, most of the Class 2 SV is in intergenic regions, not likely impacting gene expression or function.

The Class 3 deletions are particularly interesting, as they may be associated with the mutant phenotypes found in the FN irradiated individuals. For two of the FN lines, no unique deletions >40 bp were found within or near gene coding regions (although it is still possible that these lines carry heterozygous deletions within genic regions). For the remaining three FN lines, however, we identified candidate deletions within gene-encoding regions that may be associated with the resulting phenotype.

The lanceolate mutant (1R19C15r28CPVMN11) exhibited a shorter than wild type stature with elongated, or lanceolate, shaped leaves (Figure 1B). CREST analysis identified a single Class 3 SV potentially altering the expression of Phvul.001G050100 (Table 3, Figure 3). The deletion is entirely contained within the last intron region of the gene. The gene is a member of the glycosyltransferase family 47 subgroup C. Proteins encoded by members of this family are bound to the golgi and are involved in cell wall biosynthesis (Jensen et al., 2008). Altering the expression of member of this family/subgroup would impact the physical property of the pectin matrix (Jensen et al., 2008). However, there are no reports of altered leaf phenotypes or plant height in Arabidopsis knockout populations.

Three regions of SV belonging to Class 3 potentially affecting gene expression in the maturity mutant (2R29C12r78CPVMN11) were identified by CREST analysis. In the field, this plant showed no phenotypic derivation from the wild type, except a delay in maturity (Figure 1D). These three deletions, all on chromosome four, potentially affect the expression pattern of four candidate genes (Figure 3, Table 3). The 1st deletion lies immediately upstream of Phvul.004G029200, which encodes a 60S ribosomal protein. This is an unlikely candidate gene for the phenotype observed. The second deletion is in the intron of Phvul.004G031300. Sequence analysis of this gene failed to identify any functional annotations associated with this gene. The final deletion is immediately downstream of both Phvul.004G032000 and Phvul.004G031900. The Arabidopsis homologs of these genes (At3g01090 and At5g19790) encode a protein kinase and an AP2 transcription factor respectively. AP2 transcription factors are involved in regulating flowering and fruit ripening (Huijser and Schmid, 2011). In Arabidopsis, the over-expression of *AP2* results in delayed flowering and maturity (Wollmann et al., 2010). It's possible, the deletion immediately down-stream of Phvul.004G031900 causes an increase of AP2 protein accumulation resulting in delayed flowering and maturity.

Finally, the mutant plant 3R05C25r87CPVMN11 exhibited interveinal chlorosis under standard field conditions (Figure 1E). Interveinal chlorosis is often an indicator of nutrient deficiencies. However, DNA-seq analysis of this mutant revealed two SV belonging to Class 3 potentially affecting gene expression patterns (Table 3). The first is a 1518 bp deletion immediately downstream of Phvul.001G128600. The homolog of this gene in *Arabidopsis thaliana* (At3g10140) encodes a RecA protein, involved DNA repair by binding ssDNA (Miller-Messmer et al., 2012). The second Class 3 SV is a large deletion spaning 43,034 bp on chromosome 8, encompassing the entire sequence for Phvul.008G141500 (Table 3). Sequence analysis of this gene determined it is a member of the SNF2 helicase family. The *Arabidopsis homolog* of this gene, At2g44980, is the only member of the ALC1 SNF2 subfamily (Knizewski et al., 2008). There is no reported data on the phenotype of an *ALC1* knockout in Arabidopsis. However, in mammalian systems, ALC1 is essential for repairing DNA damage (Ryan and Hughes, 2011). Down-regulation of ALC1 protein results in hypersensitivity to damaging agents (Ryan and Hughes, 2011). We hypothesize a similar function is conserved in common bean. Based on the interveinal chlorotic leaf patterning when this gene is completely excised it's possible Phvul.008G141500 is involved in repairing damage to DNA in the leaves, possibly caused by UV radiation.

We identified Class 3 SV, likely a result of FN mutagenesis, ranging from 1 bp substitutions to 43,034 bp deletions, though changes <40 bp have not been visually verified. In the mutated plants, the level of FN irradiation used (16 Gys) induced far more small (<50 bp) deletions, including single base pair substitutions and deletions, than large genomic deletions. These results are consistent with FN induced deletions identified in a recent paper in Arabidopsis (Belfield et al., 2012). The lack of large SV regions belonging to Class 3, as seen in the well-characterized soybean population (Bolon et al., 2011), may be due to our analysis pipeline requiring the deletion to be complete (i.e.,: homozygous). It is possible some larger deletions are present in these lines, but are not yet homozygous in the M2 generation and, as such, were not identified by our analysis. In the soybean FN population, 52 and 38% of the mutants with visual phenotypes were identified from seeds treated with 16 and 32 Gys respectively (Bolon et al., 2011). None of the *P. vulgaris* plants irradiated at 32 Gys germinated in the field, suggesting the common bean genome may be less resilient to interruption than the soybean genome. The soybean genome may accommodate larger genomic deletions by compensating for gene loss through altered expression patterns of duplicated genes.

## Summary

A FN population with 88 individuals or bulk seed from M2 and M3 generations is available upon request. We've illustrated the utility of DNA-seq to identify three classes of SV in *P. vulgaris* individuals. These analyses were greatly facilitated by the availability of the *P. vulgaris* genome sequence. In the Red Hawk cultivar, natural variation is clustered in regions throughout the genome. These regions of natural variation illustrate the existing genetic potential of common bean germplasm. Our analyses also identified genomic deletions resulting from FN mutagenesis and candidate genes potentially responsible for the altered phenotype in three of the plants selected.

### Conflict of interest statement

The authors declare that the research was conducted in the absence of any commercial or financial relationships that could be construed as a potential conflict of interest.
